# Enzyme-Linked Aptamer Assay (ELAA) for Detection of *Toxoplasma* ROP18 Protein in Human Serum

**DOI:** 10.3389/fcimb.2019.00386

**Published:** 2019-11-13

**Authors:** Monica Vargas-Montes, Nestor Cardona, Diego Mauricio Moncada, Diego Alejandro Molina, Yang Zhang, Jorge Enrique Gómez-Marín

**Affiliations:** ^1^Centre for Biomedical Research CIBM, University of Quindío, Armenia, Colombia; ^2^Dentistry Faculty, University Antonio Nariño, Armenia, Colombia; ^3^Department of Pediatrics, Emory University, Atlanta, GA, United States; ^4^College of Science, Harbin Institute of Technology, Shenzhen, China

**Keywords:** ROP18 protein, aptamer, SELEX, ELAA, toxoplasmosis, human serum

## Abstract

*Toxoplasma gondii* engenders the common parasitic disease toxoplasmosis in almost all warm-blooded animals. Being a critical secretory protein, ROP18 is a major virulence factor of *Toxoplasma*. There are no reports about ROP18 detection in human serum samples with different clinical manifestations. New aptamers against ROP18 protein were developed through Systematic Evolution of Ligands by Exponential enrichment (SELEX). An Enzyme-Linked Aptamer Assay (ELAA) platform was developed using SELEX-derived aptamers, namely AP001 and AP002. The ELAA was used to evaluate total antigen from *T. gondii* RH strain (RH Ag) and recombinant protein of ROP18 (rROP18). The results showed that the ELAA presented higher affinity and specificity to RH Ag and rROP18, compared to negative controls. Detection limit of rROP18 protein in serum samples was measured by standard addition method, achieving a lower concentration of 1.56 μg/mL. Moreover, 62 seropositive samples with different clinical manifestations of toxoplasmosis and 20 seronegative samples were tested. A significant association between ELAA test positive for human serum samples and severe congenital toxoplasmosis was found (*p* = 0.006). Development and testing of aptamers-based assays opens a window for low-cost and rapid tests looking for biomarkers and improves our understanding about the role of ROP18 protein on the pathogenesis of human toxoplasmosis.

## Introduction

*Toxoplasma gondii* (*T. gondii*) is an intracellular parasite with cosmopolitan distribution that infects the majority of warm-blooded animals (Jones and Dubey, 2012). Nearly one third (~25%) of the world's human population may be chronically infected with *T. gondii* (Pappas et al., 2009). Infection in humans can cause severe ocular, neurologic, and sometimes systemic disease, especially in immunocompromised and congenitally infected individuals (Cardona et al., 2011; Pfaff et al., 2014). Transmission of the parasite has been demonstrated in humans by the consumption of meat, vegetables and contaminated water (Lora-Suárez et al., 2007; Franco-Hernandez et al., 2016; Triviño-Valencia et al., 2016). For all these reasons, Food and Agriculture Organization (FAO) and World Health Organization (WHO) declared toxoplasmosis as a foodborne parasite infection disease of global concern (Robertson et al., 2013).

Globally, the serological prevalence of toxoplasmosis is highly variable, ranging from 10 to 15% in the United States, to >60% in South and Central America (Gilbert et al., 2008). Additionally, it has been reported that South America is the continent with the highest burden of the disease, with congenital and ocular toxoplasmosis frequently associated with more severe symptoms (de-la-Torre et al., 2007; De-la-Torre et al., 2009; Torgerson and Mastroiacovo, 2013). The high rate of ocular toxoplasmosis in Colombia is likely attributable to exposure to more-virulent strains of *T. gondii* (Ajzenberg, 2012), even if other factors, such as inoculum exposure or the genetic background of the host, may be involved (de-la- Torre et al., 2013). Therefore, there are some indications that disease outcomes in humans can be influenced by the variability of the infecting *T. gondii* strain (Grigg et al., 2001; Reese et al., 2011; McLeod et al., 2012; Sánchez et al., 2014).

Experimental crosses between *T. gondii* strains with different virulence patterns allowed the identification of several polymorphic genes coding for secreted factors of the parasite, associated with differences in the virulence in mice (Saeij et al., 2006; Taylor et al., 2006; Talevich and Kannan, 2013). These key virulence factors include proteins from the rhoptry family (ROP kinases) that exert kinase or pseudokinase activities (Hunter and Sibley, 2012) contributing to disarm innate immunity and promote survival of the parasite (Hakimi et al., 2017). ROP18 is one of the major virulence factors of *T. gondii*, identified as a serine/threonine kinase secreted into the parasitophorous vacuole (PV) and host cytosol (Taylor et al., 2006; Talevich and Kannan, 2013). A recent study shows that ROP18 is a conserved virulence factor in genetically diverse strains from North and South America (Behnke et al., 2015). Furthermore, there is a report that demonstrates the presence of virulent alleles that code for ROP18 in humans with ocular toxoplasmosis in Colombia, who presents a more severe inflammatory reaction in the eye (Sánchez et al., 2014). Currently, there is only one study that indicates the presence of specific IgM and IgG antibodies against ROP18 in sera from humans with toxoplasmosis (Gatkowska et al., 2015). However, there are not any reported methods that allow the direct detection of this protein in human serum. ROP18 protein identification in human serum would be of great importance in order to ascertain a possible correlation between the presence of this virulent factor and the severity of the disease.

To perform the identification and quantification of protein biomarkers in serum, DNA and RNA aptamers have been used (Drolet et al., 1996; Gold et al., 2010). Aptamers are short, single-stranded oligonucleotides, that bind to targets with high affinity and specificity by folding into tertiary structures (Ellington and Szostak, 1990; Tuerk and Gold, 1990). These molecules have promising roles in clinical diagnostics and as therapeutic agents (Zhang et al., 2019), showing some advantages compared to antibodies, such as shorter generation time, lower costs of manufacturing, no batch-to-batch variability, higher modifiability, better thermal stability and higher target potential (Zhou and Rossi, 2017). Due to these characteristics, aptamers could be used as molecular recognition agents alternative to antibodies in enzyme linked immunosorbent (ELISA) assays, hence its application has given rise to the ELAA assay (Enzyme-Linked Aptamer Assay), in which aptamers are the recognition agents (Toh et al., 2015). This ELAA assay has been used to recognize *Leishmania infantum* proteins, like H2A histones (Ramos et al., 2007; Martin et al., 2013) and also for detecting *Mycobacterium tuberculosis* culture filtrate protein and secreted antigen in sputum samples from tuberculosis patients (Rotherham et al., 2012).

Although aptamer research in the area of parasitology is still in the early stages, promising results have been obtained for the main protozoan parasites, including *Trypanosoma* spp., *Plasmodium* spp., *Leishmania* spp., *Entamoeba histolytica*, and *Cryptosporidium parvuum*. These aptamers have been used to detect and treat the parasitic infections caused by these parasites in human beings (Ospina-Villa et al., 2018). For *T. gondii*, only one work with DNA aptamers has been reported for the detection of anti-*Toxoplasma* IgG antibodies (Luo et al., 2013).

**Figure 1:**
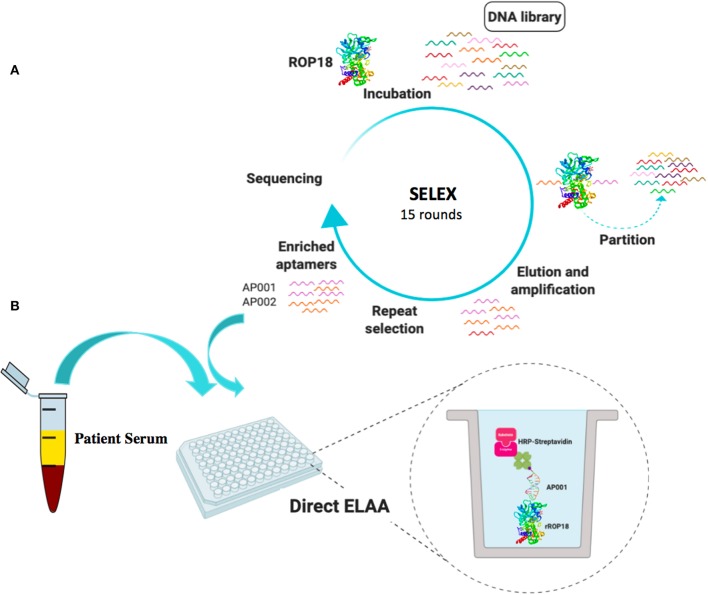
Schematic representation of SELEX process **(A)** and ROP18-ELAA platform using SELEX-derived aptamers **(B)**. This figure was created by a visual tool, BioRender.com.

There are no aptamer-based methods for the detection of *T. gondii* proteins in serum. Therefore, we developed specific aptamers against ROP18 protein by SELEX. Those newly identified aptamers were utilized in a direct or a sandwich ELAA test to detect total antigen from *Toxoplasma* and recombinant ROP18 protein. Moreover, human serum samples with rROP18 protein were analyzed, as well as the seropositive samples from individuals with toxoplasmosis were evaluated with this novel ROP18-ELAA platform (Figure 1). The newly developed aptamer-based sensing platform for ROP18, will enhance our understanding about the role of virulence factors on the pathogenesis of toxoplasmosis in humans.

## Materials and Methods

### Human Clinical Samples and Definition of Clinical Manifestations

Human serum samples for the ELAA test were obtained from 62 individuals with toxoplasmosis, 20 seronegative for the infection and 5 from individuals with a different infection as a control of specificity. Most of the samples (*n* = 67) were collected at the Center for Biomedical Research (CIBM) at the University of Quindío and some of them with ocular toxoplasmosis (*n* = 20) were recruited at the “Clínica Barraquer” in Bogotá-Colombia, with the previous signature of the informed consent. We included 18 serum samples from patients with toxoplasmic lymphadenitis (IgM and IgG anti-*Toxoplasma* positive) with avidity <50%; 13 from individuals with chronic-asymptomatic infection without eye injury (IgM anti-*Toxoplasma* negative and IgG anti-*Toxoplasma* positive); 21 from patients with ocular toxoplasmosis diagnosed by indirect ocular fundoscopy, with antibody levels positive in serum/aqueous humor (index <2), with PCR for *Toxoplasma* B1 sequence positive and based on the criteria previously described (De La Torre and López-Castillo, 2009); and 10 serum samples with congenital toxoplasmosis (IgG anti-*Toxoplasma* positive) confirmed as described by the European Network in congenital toxoplasmosis (Lebech et al., 1996). In the same way, we included 20 serum samples from seronegative individuals (IgM and IgG anti-*Toxoplasma* negative) as the negative control of the assay. Additionally, five serum samples from IgM Dengue-positive individuals (Diagnosed by an IgG capture ELISA for Dengue, Vircell Ref. M1018, carried out in the CIBM), were included to evaluate the cross-reactivity of previously standardized ELAA.

### *In vitro* Selection of Aptamers Against *T. gondii* ROP18

Two nanomoles ssDNA library (5′-ATCCAGAGTGACGCAGCA-40N-TGGACACGGTGGCTTAGT-3′) were dissolved in 350 μL binding buffer (a solution of DPBS containing 5 mM MgCl_2_, 0.1 mg/mL tRNA, 1 mg/mL BSA), mixed and heated at 95°C for 5 min, then snap cooled on ice to create folded ssDNA.

The snap-cooled DNA library was brought to room temperature and incubated with 400 pmol GST-rROP18 protein that was conjugated with Glutathione Sepharose beads at RT with rotation for 1 h. After incubation, the supernatant containing unbound sequences was removed and the beads were washed three times with 1 mL washing buffer (a solution of DPBS containing 5 mM MgCl_2_). The ssDNA-protein-bead complexes were suspended in DNase-free water for PCR amplification of ROP 18-bound sequences by using forward primer (5′-ATCCAGAGTGACGCAGCA-3′) and reverse primer with biotinylated 5′ end (5′-biotin-ACTAAGCCACCGTGTCCA-3′). PCR product was then passed three times through the DNA synthesis column loaded with streptavidin sepharose beads. The beads were washed again with 2.5 mL of PBS. 500 μL of 200 mM NaOH was added to elute the ssDNA. The eluted ssDNA was added into a NAP5 column pre-washed with 15 mL of deionized water for desalting. 1,000 μL of DNase-free water was allowed to pass through the column to elute the ssDNA. The concentration of ssDNA was determined by UV absorbance at 260 nm and concentrated by using a DNA Speedvac dryer. Precipitated ssDNA was resuspended in binding buffer for subsequent round of selection. After 15 rounds of selection, the final enriched libraries were PCR-amplified and cloned into pJET1.2/blunt cloning vector using the CloneJET PCR Cloning Kit (Thermo Fisher Scientific) according to the manufacturer's instructions. One hundred fifty Colonies from the 15th round of selection were picked and analyzed by Sanger sequencing.

### Aptamer and Antibody Anti-ROP18

Two top enriched DNA aptamers with biotin labeled were used as recognition agents. Those aptamers are AP001 with the sequence 5′-TCCTGGCAGCGCTTTTGCTTGTTTGCTCTCGTACCTGTCC-3' and AP002 with the sequence 5′-CGCACCGATCCGGTGTTAATCTCGACGTCCCTTAAGTTTG-3'. In addition, a rabbit anti-ROP18 polyclonal antibody (a gift from the Dr. L. D. Sibley from the University of Washington, Saint Louis, United States of America).

*Toxoplasma* lysate antigen from the RH strain (RH Ag) was used as positive control, because it expresses the ROP18 protein (Supplementary Figure S1). RH Ag was prepared as previously reported (Torres-Morales et al., 2014) with some modifications. Briefly, *T. gondii* tachyzoites from the RH strain were maintained *in vitro* in human fibroblasts (HFF) at 37°C and 5% CO_2_. The antigen was obtained after recovering the tachyzoites from the culture and centrifuged at 3,000 rpm for 5 min in RPMI medium, the tachyzoites pellet was resuspended in saline and subjected 5 times to freeze-thawing and to breakage by sonication 8 times a 20 W for 20 s. Subsequently, the lysis of the parasite was verified by microscopy. Finally, 1x protease inhibitor cocktail (dilution 1:100) was added to the antigen (Ref. I3786, Sigma-Aldrich, St. Louis, USA), the aliquots were performed and stored at −80°C. The protein quantification was performed by the Bicinchoninic Acid Protein Assay (Ref. 23227, Thermo Scientific, Rockford, IL) by using the spectrophotometer EPOCH (BioTek Instruments, Winooski, VT, USA) at 280 nm. The RH Ag, was evaluated at different concentrations (from 200 to 6.25 μg/ml) in order to determine the detection limit for each assay.

In addition to RH Ag, the recombinant protein ROP18 (rROP18) of *T. gondii* RH strain produced in our lab was also used as positive control in the last steps of the standardization. In the same way, three negative controls were included: the recombinant protein Disulfide isomerase of *T. gondii* (PDI); Lucifensin-CPD, a recombinant protein from the fly *Lucilia sericata* (LucGT), both produced in our lab, and bovine serum albumin (BSA) (AMRESCO), these controls were used at a concentration of 6.25 μg/mL. Likewise, a lysate antigen from a Knockout strain for ROP18 (KOROP18, a gift from the Dr. Sibley, St. Louis, USA) of *T. gondii* was used as another negative control of the assay, this antigen was prepared similar to RH Ag.

### Enzyme-Linked Aptamer Assay (ELAA) Standardization

For standardization of the ELAA assay, two different configurations were evaluated: direct and sandwich ELAA (Toh et al., 2015), in order to determine which configurations allowed to reach a higher detection limit of RH Ag and rROP18 protein in human serum. Initially, all the conditions for direct ELAA were standardized and based on these conditions we performed the sandwich ELAA, in which the only additional step was the anti-ROP18 antibody, added at the beginning of the assay.

To standardize the general protocol, the antigens (Ag RH, rROP18, PDI, LucGT, BSA, and KOROP18) were immobilized in 96-well microtiter plates (NUNC) diluted in 0.1 M carbonate buffer at a pH of 9.6 (Na_2_CO_3_, 0.159 g/100 mL; NaHCO_3_, 0.293 g/100 mL) and adding 100 μL per well. The antigen incubation was evaluated for 1 h at 37°C, or overnight at 4°C as previously reported (Rotherham et al., 2012; Luo et al., 2013). After coating, we performed 5 washes with 0.01 M phosphate buffered saline (PBS) (pH 7.4) plus 0.05% Tween 20 (PBS-T), previously used in other studies (Martin et al., 2013; García-Recio et al., 2016). Then, three different conditions were included for the blocking step: 1% BSA (AMRESCO) diluted in PBS-T (Luo et al., 2013), 5% skimmed milk (Rotherham et al., 2012) in PBS-T and no blocking, as reported in other studies (Martin et al., 2013; García-Recio et al., 2016). Each well was blocked with 300 μL of the blocking solution by 1 h at 37°C. After washing three times, biotinylated aptamers (200 nM) against ROP18 were added to each well, these oligonucleotides were diluted in binding buffer (PBS, 0.5% glucose, 0.1% albumin and 1 M MgCl_2_) and incubated for 1 h at 37°C. Then, 5 washes were performed and 100 μL of streptavidin-horseradish peroxidase conjugate (Thermo-Fisher) was added, evaluating three previously reported dilutions, 1:10,000 (Murphy et al., 2003), 1:15,000 (Rotherham et al., 2012), and 1:20,000 (Balogh et al., 2010) diluted in PBS and 1% BSA for 1 h at 37°C. Finally, after five washes, horseradish peroxidase activity was detected by using TMB for 15 min at room temperature and stopped by adding a 5% of sulfuric acid (H_2_SO_4_). The absorbance at 450 nm was read in an Epoch 2 spectrophotometer (BioTek Instruments, Winooski, VT, USA). All the samples were processed in triplicate.

### Aptamer Concentration and Binding Affinity of AP001 and AP002

In order to study the binding affinity of aptamers AP001 and AP002, 50 μg/mL (5 μg/well) of RH Ag expressing ROP18 protein were plated in coating buffer and incubated in a 96-well microtiter plate overnight at 4°C. Then, the wells were washed 5 times in PBS-T and then blocked 1 h with 1% BSA in PBS. Afterwards, three washes were performed and biotin-labeled aptamers were diluted in binding buffer at concentration between 50 and 500 nM, and then incubated at 37°C for 1 h. Next, 100 μL of streptavidin-HRP (1:10,000 dilution) were added to the individual wells and developed using TMB solution as above. Data were analyzed using non-linear regression with an equation y = (x × Bmax) / (x + Kd), where Bmax is the maximal binding and Kd is the concentration of ligand required to reach half-maximal binding.

### Detection Limit of rROP18 Protein by Direct ELAA

To identify the detection limit of the rROP18 protein in serum, concentrations from 50 to 1.56 μg/mL of the antigen were evaluated. RH Ag and KOROP18 Ag were included at a concentration of 50 μg/mL (the maximum concentration used for rROP18). All the antigens were diluted in a serum sample from a seronegative individual (IgM and IgG *Toxoplasma* negative). To select the serum dilution we analyze results of absorbance after performing ELAA protocol with 1:2, 1:5, and 1:10 dilutions of serum from one seronegative individual (IgM and IgG *Toxoplasma* negative) that was artificially spiked with 2.5 μg of recombinant ROP18 protein. The 1:10 serum dilution was the only one that allowed to differentiate between the absorbance levels of rROP18 and KOROP18 Ag (*p* = 0.022) and between rROP18 and serum without antigen (*p* = 0.023). The direct ELAA was performed with the general protocol previously standardized and only with one of the selected aptamers (AP001).

### Aptamer-Antibody Assay: Sandwich ELAA

The aptamer-antibody assay binding was performed using the direct ELAA described above with minor modifications. The anti-ROP18 polyclonal antibody was coated onto a 96-well microtiter plate overnight, diluted 1:500 in carbonate buffer and incubated overnight at 4°C. After washing five times with PBS-T, unspecific ligand sites were then saturated with 300 μL of 1% BSA diluted in PBS for 1 h at 37°C. After 3 washes, the samples were included: rROP18 was added at concentrations from 50 to 1.56 μg/mL, in order to identify a new detection limit, RH Ag and KOROP18 Ag were included again at a concentration of 50 μg/mL. All the antigens were diluted in the seronegative serum sample previously indicated and were diluted 1:10 in carbonate buffer. The samples were incubated by 2 h at 37°C with shaking. The biotinylated aptamer was then added at 300 nM in binding buffer, followed by the HRP-conjugated streptavidin (1:10,000). The detection limit obtained from this assay was compared with that obtained in the direct ELAA, with the aim to analyze if the detection limit of the protein was affected in the presence of the antibody.

### ROP18-ELAA in Human Serum Samples

The standardized direct ELAA was applied for ROP18 detection in all the serum samples previously described (*n* = 87). The 20 serum samples from seronegative individuals (IgM and IgG anti-*Toxoplasma* negative) were used to calculate the cut-off point of the test (Cut off: average absorbance plus two standard deviations). In order to normalize the data and establish a Reactivity index (RI) for each serum, the mean absorbance of each sample was divided by the cut-off point of the test. Serum samples with IR> 1 were considered positive (Caballero-Ortega et al., 2014). The serum samples were processed in duplicate and two tests were performed per sample. The inter- and intra-assay coefficient of variation [CV = (standard deviation of the RI/arithmetic mean of the RI) ^*^ 100] was calculated.

### Bioethical Aspects

This study was conducted according to the tenets of the Declaration of Helsinki, strictly following the Guide for Good Laboratory Procedures. Informed written consent, according to the regulation 008430 of 1993 of the Ministry of Health in Colombia was obtained from all people that accepted to participate in the study. The protocol was approved by the Institutional Ethical Committee (Reference numbers: 5–14-1 from Universidad Tecnológica de Pereira and 030314 from Escuela Superior de Oftalmología Instituto Barraquer de América) approved the study.

### Statistical Analysis

Data from ELAA standardization were expressed as means ± SEM. Differences in means were compared by the Student *t* test or a by non-parametric test if values were not normally distributed. Kruskal Wallis test and the Dunn test, were used for multiple comparisons between the standardization conditions. Spearman correlation test was performed to evaluate associations between quantitative variables of the population and the Reactivity index from the ELAA test. These data were analyzed using Graph Pad Prism 6.0 software (San Diego, CA, USA).

Differences in proportions between groups of patients were analyzed using the Fisher exact test. In addition, the association between the test positivity and different clinical characteristics related to the severity of ocular and congenital toxoplasmosis were evaluated. Epi-Info software 7.0 (Centers for Disease Control and Prevention, Atlanta, Georgia) was used to perform these analysis (available at: http://www.cdc.gov/epiinfo/). A *p* < 0.05 was considered to be statistically significant.

## Results

### *In vitro* Selection of ROP18 Aptamers by SELEX

A random ssDNA library was used to select aptamers binding to rROP18. GST-rROP18 protein conjugated with Glutathione Sepharose beads was used as the target. Following incubation, the bound aptamers were separated from unbound ones, and target-bound ssDNA were eluted and enriched at each round of selection by amplification using PCR. A total of 15 rounds of repeated separation-amplification cycles were completed in order to receive high affinity and specificity of DNA aptamers against ROP18 protein. Cloning and sequencing of aptamer pools from the 15 rounds of cycles identified several aptamer candidates (Figure 1A). Aptamers AP001 with the sequence 5′-TCCTGGCAGCGCTTTTGCTTGTTTGCTCTCGTACCTGTCC-3' and AP002 with the sequence 5′-CGCACCGATCCGGTGTTAATCTCGACGTCCCTTAAGTTTG-3' were the top enriched sequences, representing 14.42% and 13.46% of the final enriched population. These two novel ROP18 aptamers were labeled with biotin and utilized as biorecognition elements to construct a ELAA sensing platform. Biotin-streptavidin strategy was used for signal production (Figure 1B).

### Direct ELAA Standardization

Direct ELAA has been reported as one of the simplest and fastest methods, in which the antigen is immobilized on the surface of the platform, followed by a blocking step, addition of biotinylated aptamers, then streptavidin conjugated with HRP enzyme and the TMB substrate (Toh et al., 2015). To start with, we first developed a direct ELAA for total antigen from *Toxoplasma*. PDI, LucGT, and BSA proteins were used as negative controls. The optimal conditions of this direct ELAA test were obtained by evaluation of conditions, including time and temperature of antigen incubation, blocking solution, streptavidin dilution and aptamers concentration.

**Figure 2:**
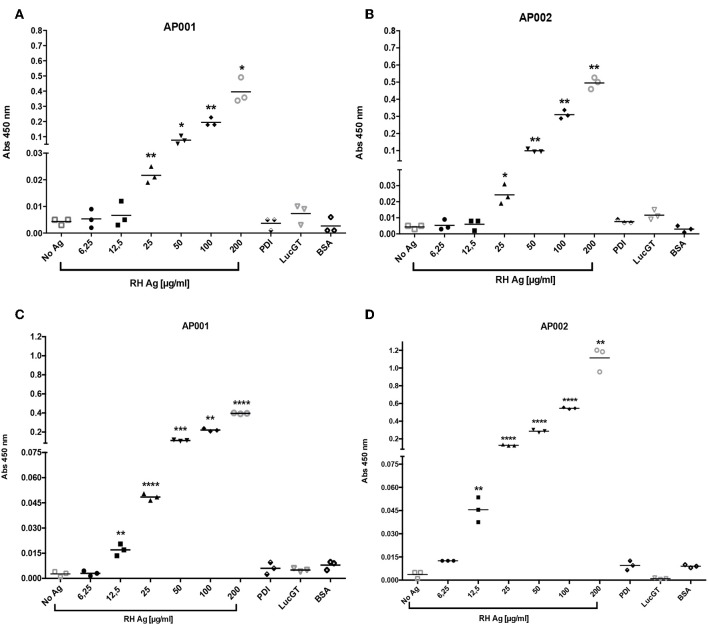
Detection limit of RH antigen according time and temperature. The ELAA assays were performed with AP001 **(A,C)** and AP002 **(B,D)** anti-ROP18 aptamers. We evaluated incubation 1 h at 37°C **(A,B)** and 4°C overnight **(C,D)**. Different concentrations of *T. gondii* total antigen of the RH strain (RH Ag) were evaluated and three negative controls, PDI, LucGT, and BSA were included. The data are represented with the mean of each sample evaluated in triplicate. Welch's *t*-test. ^*^p < 0.05, ^**^p < 0.01, ^***^p < 0.001, ^****^p < 0.0001 vs. controls without antigen (No Ag).

Firstly, we found that incubation of RH Ag overnight at 4°C allowed to reach a higher detection limit in the direct ELAA (Figure 2). Initially, antigen incubation for 1 h at 37°C was evaluated, showed that the AP001 and AP002 aptamers reached a significant antigen detection limit of 25 μg/mL compared to the negative controls (*p* < 0.05) (Figures 2A,B). Subsequently, antigen incubation was analyzed overnight at 4°C (Figure 2). We found that detection limit improved for the condition of 4°C overnight, reaching a detection limit of 12.5 μg/mL (*p* < 0.01) with both aptamers (Figures 2C,D). Overnight incubation at 4°C probably allowed more antigen adherence to the plate. Many other studies using the same incubation conditions were reported (Ramos et al., 2007; Rotherham et al., 2012; García-Recio et al., 2016).

**Figure 3:**
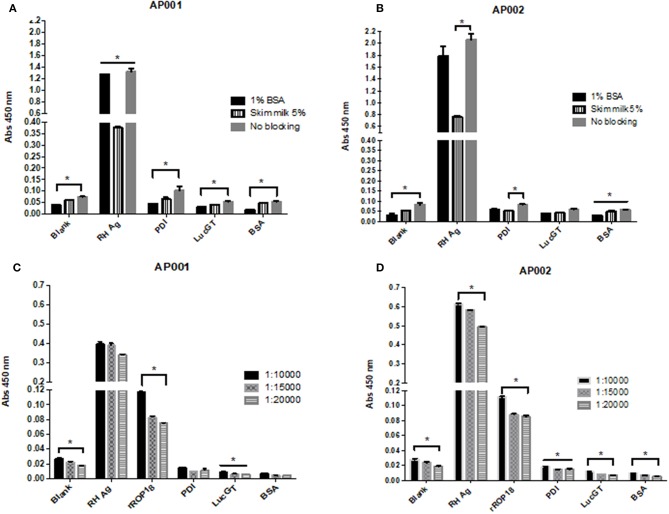
Evaluation of different blocking conditions and streptavidin dilutions for AP001 **(A,C)** and AP002 **(B,D)** ELAA test. Different blocking conditions were evaluated: 1% bovine serum albumin, 5% skim milk and no blocking **(A,B)**. Three different dilutions of streptavidin (1: 10,000, 1: 15,000 and 1: 20,000) were evaluated **(C,D)**. RH Ag was used as a positive control (50 μg/ml) for both experiments. rROP18 (25 μg/ml) was also used as positive control for streptavidin experiment. Three negative controls were included, PDI, LucGT, and BSA. The data are represented by the mean ± SEM. Kruskal Wallis. ^*^p < 0.05.

Regarding the blocking solution, we found that 1% BSA was more effective compared to the other conditions (Figure 3). For AP001 ELAA, all negative controls showed significantly lower absorbance levels for the 1% BSA condition (*p* < 0.05) (Figure 3A). In the case of AP002, lower absorbance values were detected for the negative controls with 1% BSA, although significant differences were only found for the blank condition and the negative control with albumin (Figure 3B) (*p* = 0.019 and *p* = 0.028 respectively). Regarding the positive control (RH Ag), 1% BSA and the no blocking condition allowed to reach significantly higher levels of absorbance compared to 5% skim milk condition (*p* = 0.05 and *p* = 0.03 for AP001 and AP002, respectively), indicating a higher sensitivity of the assay. However, the no blocking condition was not selected due to the non-specificity generated for the negative controls. This could explain why BSA is more effective for biotin-streptavidin systems, as it contains only one purified protein without endogenous biotin (Alegria-Schaffer et al., 2009), thus avoiding background interferences or non-specific interactions. That's probably the reason why other studies also reported the use of BSA as blocking agent for ELAA tests with biotinylated aptamers (Vivekananda and Kiel, 2006; Balogh et al., 2010; Luo et al., 2013). Therefore, we continued working with 1% BSA as a blocking agent.

Related to streptavidin dilution, we found that 1:10,000 dilution allowed to reach higher absorbance levels in the positive controls of the assay (Figures 3C,D). In AP001 ELAA, only the absorbance levels for rROP18 were significantly higher for 1:10,000 dilution (*p* = 0.021) (Figure 3C); whereas in the ELAA test with AP002, the absorbance was significantly higher for both, the RH Ag and rROP18 for 1:10,000 dilution compared to 1:20,000 dilution (*p* = 0.013 and *p* = 0.040, respectively). Regarding the negative controls, although differences between evaluated dilutions were found, mainly for AP002 (Figure 3D), the absorbance levels obtained were very low for all controls with all dilutions, with mean values of OD that ranged between 0.005 and 0.028. Therefore, considering that 1:10,000 dilution favored the sensitivity of the experiment, it was selected for the subsequent trials. This result agrees with other studies using biotinylated aptamers (Murphy et al., 2003; Rotherham et al., 2012; Stoltenburg et al., 2016).

It is worth noting that both aptamers showed a minimal recognition profile toward three negative control proteins (PDI, LucGT, and BSA), compared to the positive control (RH Ag). The significantly lower absorbance levels in negative controls suggested a higher specificity of the ELAA test.

### Aptamer Concentration and Binding Affinity of AP001 and AP002

**Figure 4:**
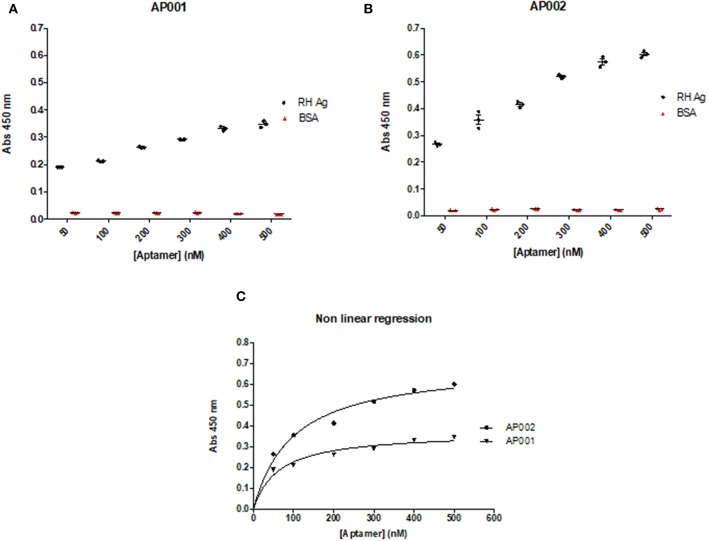
Concentration and Binding affinity of AP001 **(A)** and AP002 **(B)** aptamers. Ag RH was used as a positive control and different concentrations of each aptamer (from 50 to 500 nM) were used. BSA was used as a negative control. The dissociation constant (Kd) was calculated through a non-linear regression to define the affinity of aptamers to the RH Ag, obtaining a Kd of 62.7 nM for AP001 and a Kd of 97.7 nM for AP002 **(C)**.

A direct ELAA including all the previous standardized conditions was performed to analyze the optimal aptamer concentration. Aptamer concentrations were analyzed from 50 to 500 nM. We found that recognition of RH Ag was concentration-dependent, therefore, the absorbance levels increased as the aptamer concentrations increased (*r* = 1; *p* = 0.003; Spearman correlation test) (Figures 4A,B). The same pattern has also been found in other studies (Martin et al., 2013; García-Recio et al., 2016). Based on these results, we concluded that it was possible to continue working with an intermediate aptamer concentration (300 nM) in the subsequent ELAA tests, since it allowed an appropriate detection of the antigen, with acceptable absorbance levels (OD: 0.3–0.5) and thus allowing a moderate use of the capture reagent.

Additionally, to determine the binding affinity, we used the absorbance and concentration data from this experiment to calculate the dissociation constant (Kd). The data were analyzed using a non-linear regression, where Kd is the concentration of ligand (aptamer) required to reach half of the maximum bond, finding that a lower value of Kd will be obtained by the aptamer with greater affinity toward the antigen. Regarding this analysis, we found that aptamer AP001 showed a higher affinity with a Kd value of 62.7 ± 17.27 nM; whereas the aptamer AP002 showed a Kd of 97.7 ± 22.20 nM (Figure 4C); these results suggested that it was feasible to continue working with AP001 aptamer in subsequent trials with human serum samples.

### Detection Limit of rROP18 Protein in Serum Samples by Direct ELAA and Sandwich ELAA

**Figure 5:**
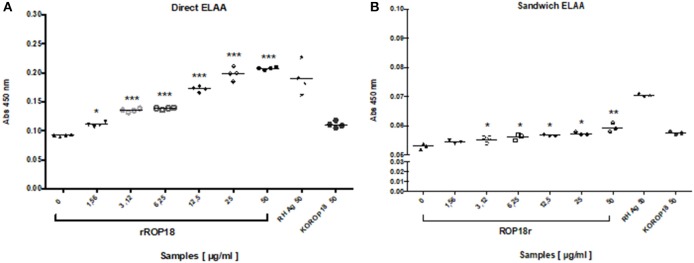
Evaluation of the detection limit of rROP18 protein in serum through direct **(A)** and sandwich **(B)** ELAA. Protein concentrations from 50 to 1.56 μg/mL diluted in serum from a seronegative individual for *T. gondii* were included. Total Ag of the RH strain (RH Ag at 50 μg / ml) was included as a positive control, and total antigen of the KOROP18 strain (KOROP18 at 50 μg / ml) was included as a negative control. The data are represented with the average of each sample evaluated in quadruplicate. Welch *t* test. ^*^p < 0.05, ^**^p < 0.01, ^***^p < 0.001 vs. the negative control KOROP18 (serum without antigen).

In order to identify the detection limit of direct ELAA with serum samples, rROP18 protein concentrations from 50 to 1.56 μg/mL were evaluated by standard addition method. The recombinant ROP 18 protein was added in the seronegative human serum sample and then diluted 1:10 in coating buffer. Total Ag of the RH strain was included as a positive control and total antigen of the KOROP18 strain was used as a negative control. The results indicated that recognition of the ROP18 protein was concentration-dependent and AP001 was able to detect rROP18 protein in serum since the minimum concentration (1.56 μg/mL), showing significant differences compared to the serum sample without antigen KOROP18 (*p* = 0.028) (Figure 5A).

In comparison, we also performed a sandwich ELAA using an anti-ROP18 polyclonal antibody as a capture agent and the aptamer AP001 as a detection agent. The data showed that sandwich ELAA allowed the detection of rROP18 protein since a concentration of 3.12 μg/mL (Figure 5B) in the serum. These results indicated that the sensitivity of sandwich ELAA was lower than the direct ELAA (1.56 μg/mL). We also found that absorbance levels obtained for sandwich ELAA were reduced, presenting OD values between 0.054 ± 0.002 for the minimum and 0.059 ± 0.001 for the maximum concentration of rROP18 protein (Figure 5B); while in the direct ELAA the absorbance values in the same concentration of the protein were 0.111 ± 0.001 and 0.207 ± 0.001, respectively (Figure 5A). Therefore, we concluded that direct ELAA was a more suitable configuration to be applied in the serum samples of individuals with toxoplasmosis.

### ROP18-ELAA Tests in Human Serum Samples From Individuals With Toxoplasmosis

To validate the suitability of the ROP18-ELAA platform on serum samples from individuals with toxoplasmosis, the direct ELAA with AP001 aptamer was applied. A total of 62 serum samples from individuals with different clinical manifestations of toxoplasmosis and 20 samples from seronegative individuals were included. The Reactivity Index (RI) was calculated for each sample. Due to the samples were processed in duplicate and two tests were performed per sample, the respective variation coefficients (VC) were calculated.

**Figure 6:**
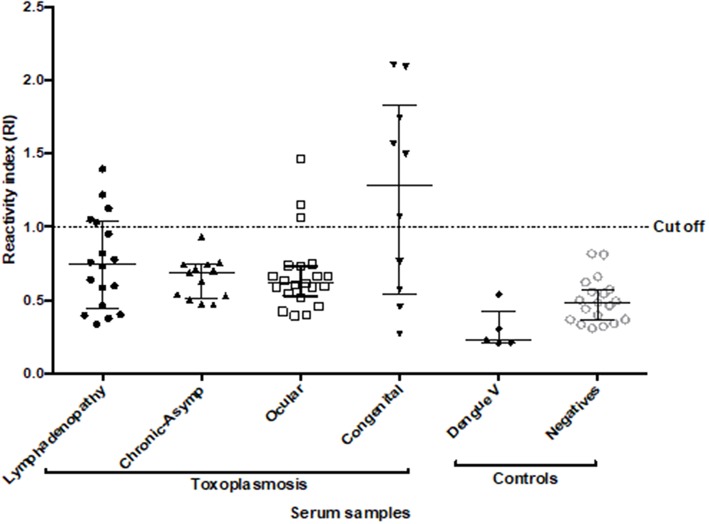
Reactivity Index values obtained with ROP18-ELAA for the human serum samples. Serum samples with different clinical forms were included: toxoplasmic lymphadenitis (*n* = 18), chronic-asymptomatic toxoplasmosis (*n* = 13), ocular toxoplasmosis (*n* = 21), and congenital toxoplasmosis (*n* = 10). Additionally, samples with Dengue virus (*n* = 5) and individuals seronegative for *Toxoplasma* (*n* = 20) were included as negative controls. Two tests were performed for each sample. The data are represented with the median and the interquartile range. Serum samples with IR > 1 are considered positive.

All the samples presented values lower than 10% for the intra-assay VC and <20% for the inter-assay VC. Due to the complexity of the human serum samples, ELAA test was positive for 22.6% (14/62) (IC95 = 13.8–34.5%) of the total samples with toxoplasmosis. We also found a positivity of 27.7% (5/18) (IC95 = 12.2–51.2%) in the group with toxoplasmic lymphadenitis, no positive samples (0/13) (IC95 = 0–26.5%) for the group with chronic-asymptomatic toxoplasmosis; 14.3% (3/21) (IC95 = 4.14–35.4%) in the group with ocular toxoplasmosis and finally 60% of positivity (6/10) (IC95 = 31.2–83.3%) for the group with congenital toxoplasmosis. Additionally, five serum samples from individuals with dengue virus were included, in which no reactivity was found for the test (Figure 6).

The comparison of RI between the group of individuals with toxoplasmic lymphadenitis and chronic-asymptomatic toxoplasmosis indicated that there were no statistically significant differences (*p* = 0.412). In the same way, although the percentage of positivity was higher in the group with lymphadenitis, no significant association was found between ELAA positivity and the acute or chronic stage of the infection (*p* = 0.058). Similarly, when comparing the RI between the groups with different clinical manifestations of toxoplasmosis, no statistically significant differences were found between them (*p* = 0.162) (Figure 6).

We found that the group with congenital toxoplasmosis had the highest RI values (Me: 1.285 Range: 0.270–2.104) and the highest positivity percentage in the test. So, the statistical analysis showed a significant association between this clinical form and the positivity for the ELAA test (*p* = 0.006). Additionally, after a stratified analysis according the clinical characteristics inside this group, we found that the positivity of the ELAA test was associated with higher severity of the disease, in other words the test was significantly positive for children with severe clinical manifestations such as presence of ocular and/or neurological symptoms than in children with congenital asymptomatic infection (*p* = 0.033, Table 1).

**Table 1:** Clinical characteristics of the relationship between population with congenital toxoplasmosis and the ELAA positivity or RI value.

**Statistical test**	**Variable (*n*)**	***p*-value**	***r***
Fisher's exact test (ELAA % positivity)	IgM positive (*n* = 10)	0.400	
	Sex (*n* = 10)	0.523	
	Symptoms (ocular and/or neurologic vs. asymptomatic) (*n* = 10)	0.033^*^	
	Neurologic symptoms (*n* = 10)	0.200	
Spearman correlation test (RI values)	Age (*n* = 10)	0.624	−0.170
	Total IgM (UI/ml) (*n* = 10)	0.441	−0.222
	Total IgG (UI/ml) (*n* = 10)	0.664	0.161

*Bold values indicates statistical significant values p <0.05*.

For the group of ocular toxoplasmosis, no statistical association was found between this clinical manifestation and ELAA positivity (*p* = 0.342). In the same way, other variables analyzed inside this group didn't show significant associations with the RI values obtained in the ELAA test; except for the total number of chorioretinal scars where we found a negative correlation (*r* = −0.74, *p* = 0.003) with the RI values (Supplementary Table S1).

Finally, some other characteristics in the total population, like age, gender, total IgM and IgG levels, as well as avidity percentage were related with the positivity of the ELAA test or the RI values; however, we didn't find any significant association between these variables (Supplementary Table S2).

## Discussion

Previous studies have reported that *T. gondii* produces some virulence factors that can modulate the host immune response and could explain the severe manifestations of toxoplasmosis, especially in South America (Bradley and Sibley, 2007; Etheridge et al., 2014; Petersen et al., 2017). The ROP18 protein has been described as one of the major virulence factors of *T. gondii*, involved in the regulation of the host innate immune response, promoting the survival and replication of the parasite (Saeij et al., 2006; Taylor et al., 2006). IgM and IgG antibodies have been identified against the ROP18 (Gatkowska et al., 2015) or against peptides derived from it (Sánchez et al., 2014), which indicates that the immune system recognizes the protein. However, until now, the presence of the protein in serum from individuals with toxoplasmosis has not been reported and it is unknown if its presence could be related to the clinical manifestation of the disease.

Although antibodies to detect ROP18 protein are available, are difficult to obtain in developing countries and there are no other tools to readily and routinely assess *T. gondii* protein in serum. Aptamers are nucleic acids that are capable of selective binding to targets of interest. In addition to the easiest and cheaper production, the use of aptamers as biorecognition tools have several advantages in terms of storage compared to antibodies. Therefore, development and testing of aptamers-based technology for *T. gondii* protein opens a window for low-cost and rapid diagnostics that could in part support the great demand for point-of-care diagnostics in developing countries.

In this study, we developed DNA aptamers against ROP18 from *T. gondii* by the SELEX method. By utilizing those newly enriched aptamers, we developed a novel aptamer based biosensing platform for serum samples from people with toxoplasmosis. A direct ELAA was initially evaluated using recombinant protein ROP18 (rROP18) and total antigen from *T. gondii* RH strain. The optimal conditions, including time and temperature of incubation, as well as the buffer composition and aptamer concentration were achieved, allowing a best detection performance. Additionally, we found that AP001 was the aptamer with the higher affinity against the antigen.

The detection limit of the direct ELAA with aptamer AP001 was evaluated with rROP18 diluted in human serum samples. Similarly, we developed a sandwich ELAA configuration in order to compare which configurations allowing a greater sensitivity. The results indicated that direct ELAA was more sensible, allowing the detection of the protein in serum since a concentration of 1.56 μg/mL, while the sandwich configuration showed a detection limit of 3.12 μg/mL. Considering those results, we used the direct ELAA to analyze the serum samples from individuals with different clinical manifestations of toxoplasmosis. Our results indicated that the presence of the ROP18 protein was found significantly in higher proportion in serum from people with congenital toxoplasmosis group, but also, they had the highest RI values compared with the other groups. These data suggested that the group of congenital individuals may present a higher parasitic load and therefore a possible secretion of ROP18 proteins at higher levels. Also, it could be explained because the immune response generated in these individuals is not as efficient to control the infection caused by the pathogen as occurs in other clinical forms. It has been described that clinical manifestations in congenital infection are related to a host genetic susceptibility that lead to an insufficient control of the parasite compared to children also congenitally infected but without symptoms (Jamieson et al., 2010).

Additionally, we found interesting association between the presence of ocular and/or cerebral symptoms in the group with congenital toxoplasmosis and the positivity of ELAA test; therefore, the presence of ROP18 could be suggested as a biomarker related to a greater severity in this clinical form. On the other hand, although no statistically significant association was found between the acute or chronic stage of toxoplasmosis and the positivity of the ELAA test, we observed a tendency of higher percentage of positivity and elevated RI values in the group of individuals with toxoplasmic lymphadenitis. This result could suggest that individuals with the acute stage of the infection and the presence of *T. gondii* tachyzoites in blood (Halonen and Weiss, 2013) have more probability to be secreting the ROP18 protein. In support of this assumption, we found a negative correlation with the number of chorioretinal scars, that could be explained because increased number of scar indicates longer time from acquisition that it is related to the number of recurrences in one individual (De-la-Torre et al., 2009). Likewise, in chronic-asymptomatic individuals (negative for the ELAA assay) the absence of the ROP18 protein could be explained by the chronic stage of the infection, in which the parasite is found in a dormant stage called bradyzoite, which is slow growing and it is controlled by the host's immune system (Blader and Saeij, 2009).

Importantly, we didn't find positivity in the ELAA test with serum samples from individuals with dengue virus, which indicated that the test was specific and did not detect antigens from another pathogenic agent. However, it is important to evaluate more serum samples with other parasitic diseases such as malaria and leishmaniosis, as well as with other viral and bacterial infections.

A relevant fact of the present study is the explanation of how the ROP18 protein of *T. gondii* reaches the serum of individuals with toxoplasmosis. Previous studies indicate that ROP18 is secreted by the rhoptry organelles inside the host cell during the process of parasite invasion and later it is located in the membrane of the parasitophorous vacuole (Saeij et al., 2006; Hunter and Sibley, 2012). However, it is possible to suggest that the parasite secretes a certain amount of the ROP18 protein before entering to the host cell and it also could explain the presence of IgM and IgG antibodies in mouse and human serum that recognize specifically the ROP18 protein (Gatkowska et al., 2015; Grzybowski et al., 2015). Additionally, it could be assumed that the ROP18 protein is secreted once the parasite has been established within the host cell. A recent study shows that the secretion of proteins by the microneme organelles is directed by *in vitro* exposure to serum albumin, a host protein (Brown et al., 2016). A similar event could occur with the rhoptry organelles, being stimulated by any host protein to secrets some ROP kinases. Furthermore, we can propose that the ROP18 protein is released after the disruption of the host cell, caused by the uncontrolled replication of the parasite. This cellular breakdown has been reported mainly by infection with type I virulent strains in mice, which are not effectively controlled by the immune system of this murine host (Melo et al., 2011).

In conclusion, two ROP18-aptamers were selected by a SELEX method and were used to standardize an ELAA test. Results showed that AP001 aptamer had a higher affinity for rROP18 and RH *T. gondii* antigen, and therefore it was used to detect ROP18 in serum samples from people with different clinical forms of toxoplasmosis. The ELAA test with AP001 was positive in 60% of people with congenital infection and in 22.6% of the cases with toxoplasmosis. These results suggest that ROP18-ELAA could be used as a potential test to identify severity of the congenital toxoplasmosis. One limitation of this study is that were analyzed only one sample per patient, and it would be important to have a longitudinal follow up in order to identify how is the variation in levels of ROP18 according evolution of symptoms and the effect of treatment. This should be analyzed in a future study. Present findings open new research avenues to understand the role of virulence factors of *T. gondi* on the pathogenesis of toxoplasmosis in humans.

## Data Availability Statement

The datasets generated for this study are available on request to the corresponding author.

## Ethics Statement

The studies involving human participants were reviewed and approved by the Institutional Review Board from Universidad Tecnológica de Pereira. Written informed consent to participate in this study was provided by the participants' legal guardian/next of kin.

## Author Contributions

MV-M: conceptualization, methodology, validation, formal analysis, investigation, and writing-original draft. NC: conceptualization, methodology and formal analysis, and writing-original draft. DAM: methodology, analysis and formal analysis. DMM: conceptualization, methodology, and formal analysis. YZ: conceptualization, methodology, and writing-review and editing. JG-M: conceptualization, writing-reviewing and editing, supervision.

### Conflict of Interest

The authors declare that the research was conducted in the absence of any commercial or financial relationships that could be construed as a potential conflict of interest.
