# Supplementary material for: Enzyme-Linked Aptamer Assay (ELAA) for Detection of Toxoplasma ROP18 Protein in Human Serum
Source: Front Cell Infect Microbiol. 2019 Nov 13;9:386. doi: 10.3389/fcimb.2019.00386 (PMC6863806; doi:10.3389/fcimb.2019.00386)
Supplement: Supplementary file 1 [file Data_Sheet_1.docx]

Supplementary Material

**Supplementary Figures**

**RH Ag**

**Af**

**BSA**

**Af**

**MP**

**Af**


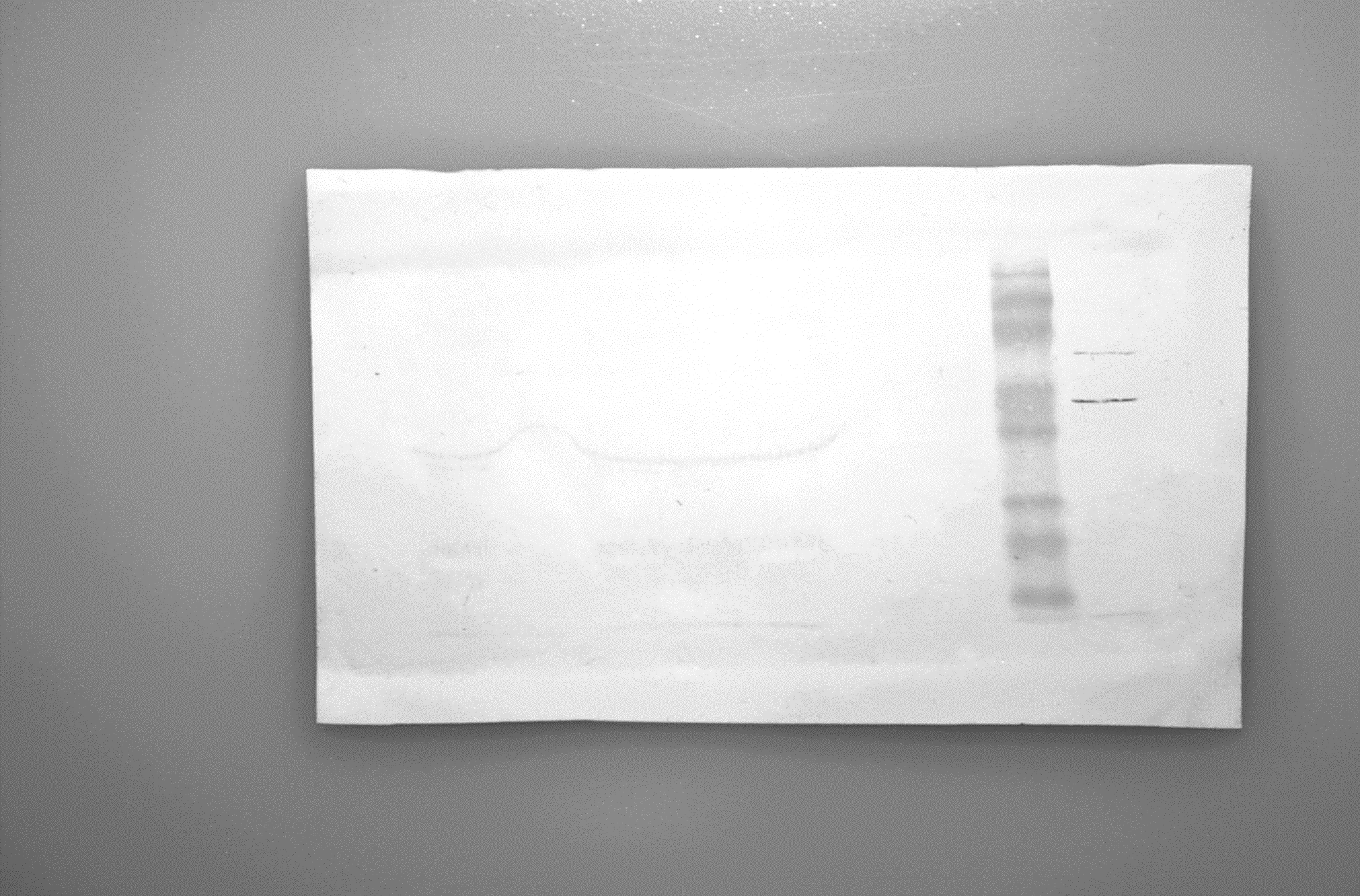

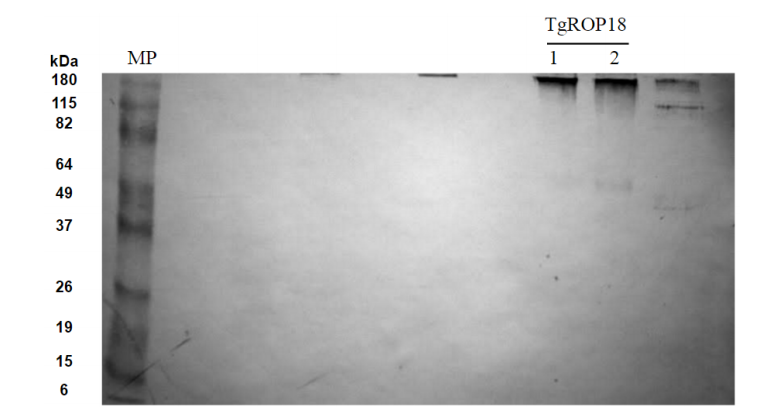


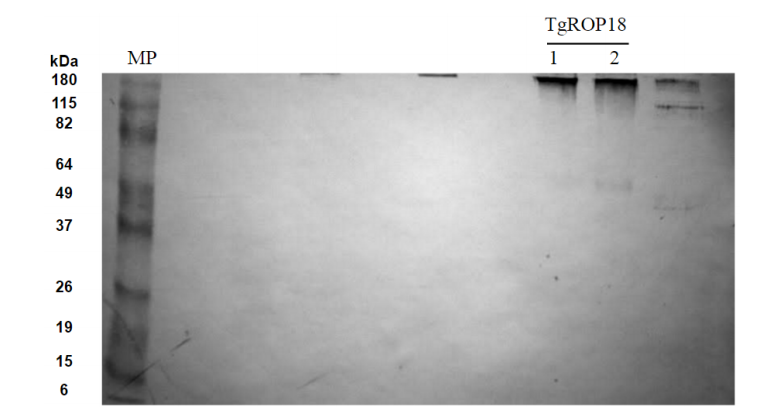


**Supplementary Figure S1.** Western Blot to detect the ROP18 protein in total antigen from *Toxoplasma gondii* RH strain (20μg) (RH Ag). MP. Molecular weight marker BenchMark ™ PreStained. BSA was used as a negative control (20μg).

# Supplementary Tables

**Supplementary Table S1.** Correlation between the population with ocular toxoplasmosis and ELAA positivity or RI values. RE: right eye, LE: left eye, AH: aqueous humor, VH: vitreous humor.

| **Statistical test** | **Variable (n)** | **p value** | **r** |
| --- | --- | --- | --- |
| **Fisher's exact test** (ELAA % positivity) | Bilateral retinal scars (n=18) | 0.686 |  |
|  | GRA 6-I serotype (n=18) | 0.294 |  |
|  | PCR-B1 in AH. (n=18) | 0.294 |  |
|  | IgM positive (n=21) | 0.386 |  |
| **Spearman correlation test** (RI values) | Total number of chorioretinal scars (n=13) | 0.003 | -0.737 |
|  | Active lesion size RE (n=11) | 0.182 | -0.407 |
|  | Active lesion size LE (n=13) | 0.239 | 0.351 |
|  | Number of recurrences (n=16) | 0.711 | 0.099 |
|  | Level of inflammation in AH  (n=16) | 0.357 | 0.245 |
|  | Level of inflammation in VH (n=15) | 0.680 | 0.115 |
|  | Avidity % (n=20) | 0.215 | -0.289 |
|  | Total IgM (UI/ml) (n=21) | 0.304 | -0.35 |

**Supplementary Table S2.** Correlation between the subpopulation of toxoplasmosis and ELAA positivity or RI values.

| *Statistical test* | *Variable*  *(n)* | *p value* | *r* |
| --- | --- | --- | --- |
| Fisher's exact test (ELAA positivity) | Gender (n=62) | 0.618 |  |
|  | IgM positive  (n=62) | 0.553 |  |
| Spearman correlation test (RI values) | Age  (n=60) | 0.073 | -0.233 |
|  | Total IgM (UI/ml) (n=21) | 0.493 | -0.089 |
|  | Total IgG (UI/ml) (n=21) | 0.349 | 0.121 |
|  | Avidity % (n=20) | 0.568 | 0.082 |
